# 烯醇化酶ENO1抑制非小细胞肺癌细胞上皮间质转换

**DOI:** 10.3779/j.issn.1009-3419.2013.05.01

**Published:** 2013-05-20

**Authors:** 鑫 周, 莹 张, 廼珺 韩, 素萍 郭, 汀 肖, 书钧 程, 燕宁 高, 开泰 张

**Affiliations:** 1 100021 北京，北京协和医学院，中国医学科学院，肿瘤医院肿瘤研究所，分子肿瘤学国家重点实验室，癌发生及预防分子机理北京市重点实验室 State Key Laboratory of Molecular Oncology, Beijing Key Laboratory of Carcinogenesis and Cancer Prevention, Cancer Institute (Hospital), Chinese Academy of Medical Sciences and Peking Union Medical College, Beijing 100021, China; 2 400016 重庆，重庆医科大学生物化学与分子生物学教研室，医学分子肿瘤研究中心 Department of Biochemistry and Molecular Biology, Molecular Medicine and Cancer Research Centre, Chongqing Medical University, Chongqing 400016, China

**Keywords:** ENO1, 肺肿瘤, 上皮间质转换, ERK1/2, ENO1, Lung neoplasms, Epithelial-mesenchymal transition, ERK1/2

## Abstract

**背景与目的:**

已有的研究表明：上皮间质转换（epithelial-mesenchymal transition, EMT）是非小细胞肺癌发展和转移的一个重要过程，受到众多信号通路的精细调节。经典的丝裂原活化激酶（mitogen activated protein kinase, MAPK）信号通路是转化生长因子（transforming growth factor β, TGFβ）诱导EMT发生的必要条件。本研究以非小细胞肺癌细胞系A549为模型，对烯醇化酶（enolase-1, ENO1）影响细胞EMT过程的分子机制进行了初步研究。

**方法:**

建立稳定过表达ENO1的A549细胞，用划痕实验检测细胞运动能力；用Western blot技术检测EMT过程相关分子标志物的变化；通过TGFβ-1诱导实验检测ENO1过表达对EMT的影响；通过上皮生长因子（epidermal growth factor, EGF）诱导实验和Western blot检测ENO1过表达引起胞外信号调节激酶（extracellular signal regulated protein kinase, ERK）磷酸化的改变。

**结果:**

ENO1过表达抑制A549细胞侧向迁移能力。ENO1过表达还会引起上皮样标志物E-cadherin表达上调，同时间质样标志物N-cadherin和Vimentin表达下降；TGFβ-1诱导实验也证实了ENO1对EMT进程的抑制作用。EGF活化实验显示ENO1对ERK磷酸化的抑制作用。

**结论:**

在非小细胞肺癌细胞中，ENO1具有抑制细胞EMT的作用，且很可能是通过抑制MAPK通路来实现。

## Abstract (en)

**Background and objective:**

It has been proven that epithelial-mesenchymal transition (EMT) is a critical process which is precisely regulated by multiple signaling pathways during the progression and metastasis of non-small cell lung cancer (NSCLC). Canonical MAPK signaling is essential to transforming growth factor β (TGFβ)-induced EMT. Using the NSCLC cell line A549 as a model, the aim of this study is to explore the molecular mechanism of ENO1 affecting EMT.

**Methods:**

We established an A549 strain stably overexpressing ENO1. Cell mobility was measured by the wound-healing assay. EMT-related molecular alterations were detected by Western blot analysis. The effect of ENO1 on EMT was also detected by TGFβ-1-inducing assay. EGF-stimulating assay was performed to illustrate ERK1/2 phosphorylation changes resulting from ENO1 overexpression.

**Results:**

Overexpressed ENO1 inhibited the mobility of A549 (*P* < 0.05), as well as the expression of the mesenchymal markers N-cadherin and vimentin, but upregulated the epithelial marker E-cadherin. TGFβ-inducing assay also showed that the negative effect of ENO1 on EMT. ERK1/2 phosphorylation was also obviously suppressed by ENO1 in the EGF-stimulating assay.

**Conclusion:**

In NSCLC cells, ENO1 overexpression can inhibit EMT *in vitro* by suppressing ERK1/2 phosphorylation.

Elizabeth Hay于1968年提出上皮间质转换的概念，并指出这种转换在一定条件下是可以逆转的^[1]^。事实上，EMT过程在后生动物的整个生命周期中都起到重要作用，如发育、组织损伤修复、纤维化，以及某些病理过程（如恶性肿瘤的转移）^[2]^。最新的统计^[3]^显示，肺癌死亡率仍高居各类癌症之首。非小细胞肺癌（non-small cell lung cancer, NSCLC）主要起源于上皮组织，因此大部分癌细胞维持上皮样特性，小部分细胞会通过EMT获得成纤维细胞样形态，失去胞间粘附并获得移动能力^[4]^。在分子水平上，这部分间质样细胞通常丢失E-钙粘蛋白（E-cadherin）、桥粒斑蛋白（Desmoplakin）和紧密连接蛋白（zonula occluden, ZO-1）等上皮样细胞表面分子，而获得间质样细胞标志如N-钙粘蛋白（N-cadherin）和波形蛋白（Vimentin），伴随着相关转录因子Twist、Snail和Slug的表达上调^[5]^。EMT是包含一系列精细复杂的变化，多种信号通路都参与其中，主要包括Wnt信号通路、TGFβ信号通路、Notch信号通路、Hedgehog信号通路、磷脂酰肌醇激酶（phosphatidyl inositol 3-kinase, PI3K）信号通路、核因转录因子（nuclear factor-κB, NF-κB）信号通路和ERK信号通路等；此外，还有多种microRNA参与这一过程的调控，如miR-155、miR-200和miR-205^[6, 7]^。

除了肺癌，在诸如结肠癌^[8]^、胃癌^[9]^和乳腺癌^[10]^等多种实体瘤的研究中，都发现发生EMT的肿瘤细胞具有更高的恶性程度。并且，原发灶的肿瘤细胞出现EMT，常常预示着较差的临床预后。一项针对EMT相关转录因子Slug的临床研究^[11]^发现，肺癌组织中其mRNA水平越高，患者的术后复发几率越高，生存期越短。

相比于正常组织，肿瘤组织即使在有氧条件下糖酵解过程也明显加强，并且相关酶类活性和表达水平都有所提高，即Warburg效应^[12]^。随后更多研究发现，糖酵解过程相关酶类如己糖激酶（hexokinase-2, HK-2）、乳酸脱氢酶（lactate dehydrogenase-A, LDH-A）、甘油醛-3-磷酸脱氢酶（glyceraldehyde-phosphate dehydrogenase, GAPDH）和ENO1，实际上是具有包括催化活性在内的多功能蛋白^[13]^。编码ENO1的mRNA可以通过选择性翻译，表达另一种短异构体c-Myc启动子结合蛋白1（c-Myc promoter binding protein-1, MBP-1）^[14]^。多种证据表明在乳腺癌^[15]^、NSCLC^[16]^、丙肝病毒相关肝细胞肝癌^[17]^、前列腺癌^[18]^和神经胶质瘤^[19]^中，ENO1和MBP-1都参与了癌症的发展过程。在人乳腺癌细胞MCF-7中过表达MBP-1，可以抑制细胞的侵袭能力^[15]^。在胃癌中，ENO1和MBP-1可以通过抑制环氧合酶（cyclooxygenase-2, COX-2）表达抑制细胞EMT过程，从而抑制细胞侵袭和转移能力^[20]^。目前，ENO1对肺癌细胞EMT的影响，没有明确阐述。

本研究旨在揭示ENO1对NSCLC细胞系A549的EMT过程的影响，及其潜在的分子机制。

## 1 材料与方法

### 1.1 野生型和突变型*ENO1*基因的克隆和真核表达质粒的构建

以人胚胎cDNA文库作为模板，利用LATaq聚合酶（Takara，日本）克隆野生型ENO1全长；并构建至pcDNA^TM^3.1/myc-His(-)A载体（Invitrogen，美国）中。随后，利用PCR引入点突变的方法，将野生型ENO1第94和97位氨基酸对应密码子ATG突变为CTG。突变型ENO1m同样构建于pcDNA^TM^3.1/myc-His(-)A载体中。目的片段序列测定由上海生工生物工程公司完成。

### 1.2 细胞培养、脂质体介导的细胞转染

人NSCLC细胞系A549（ATCC，美国），在含有10%胎牛血清的RPMI-1640（Invitrogen，美国）完全培养基中置于5%CO_2_、37 ℃条件下培养。当细胞融合度达到90%时进行质粒转染，按照Lipofectamine^TM^2000（Invitrogen，美国）产品说明书操作。转染质粒24 h后，替换含终浓度为600 µg/mL G418的完全培养基中进行筛选，并维持选择压力，从而获得稳定过表达ENO1的细胞亚群，命名为A549-ENO1。对照组转染空载质粒，命名为A549-Ctrl。

### 1.3 划痕实验

当细胞融合度达到100%时，在培养皿中均匀划出三道划痕。磷酸盐缓冲液洗涤去除划下的细胞。加入含2%胎牛血清的RPMI-1640培养基，于37 ℃、5%CO_2_条件下培养，此时记为第0天，并拍照记录。随后，每隔24 h更换含2%胎牛血清的培养基并拍照记录。

### 1.4 TGFβ-1诱导上皮间质转换实验

当细胞融合度在90%时，吸尽培养基，并用磷酸盐缓冲液洗涤。细胞经无血清培养饥饿24 h后，添加指定浓度TGFβ-1（R & D，美国）处理24 h，并拍照记录。

### 1.5 EGF刺激实验

当细胞融合度在50%-70%时，吸尽培养基，并用磷酸盐缓冲液洗涤。细胞经无血清培养基饥饿4 h后，加入终浓度为20 ng/mL的EGF（Cell Signal，美国）处理1 h。

### 1.6 细胞总蛋白的提取和Western blot分析

以适量含有1%苯甲基磺酰氟（PMSF）、1%蛋白酶抑制剂和1%磷酸酶抑制剂的RIPA蛋白裂解液（普利莱，中国）提取细胞总蛋白。采用BCA Protein Assay Kit（Thermo，美国）进行蛋白定量后用于Western blot分析。所用主要抗体及稀释比：ENO1（1:1, 500，奥维亚，中国）、ERK1/2（1:2, 000，Santa Cruz，美国）、p-ERK1/2（1:1, 000，Santa Cruz，美国）、p-MEK1/2（1:1, 000，Cell Signal，美国）、E-cadherin（1:1, 000，Santa Cruz，美国）、N-cadherin（1:1, 000，BD，美国）、Vimentin（1:500，Santa Cruz，美国）和β-actin（1:5, 000，Santa Cruz，美国）。

### 1.7 统计学分析

细胞划痕实验结果图采用ImageJ2X（National Institutes of Health）采集数据，SPSS Statistics 17.0进行两组独立样本*t*检验，*P* < 0.05为差异有统计学意义。

## 2 结果

### 2.1 筛选稳定过表达ENO1的A549细胞

收集筛选后的A549-ENO1和对照A549-Ctrl总蛋白，进行Western blot检测。结果显示，相比于对照组，A549-ENO1的ENO1表达水平更高（图 1）。

**1:**
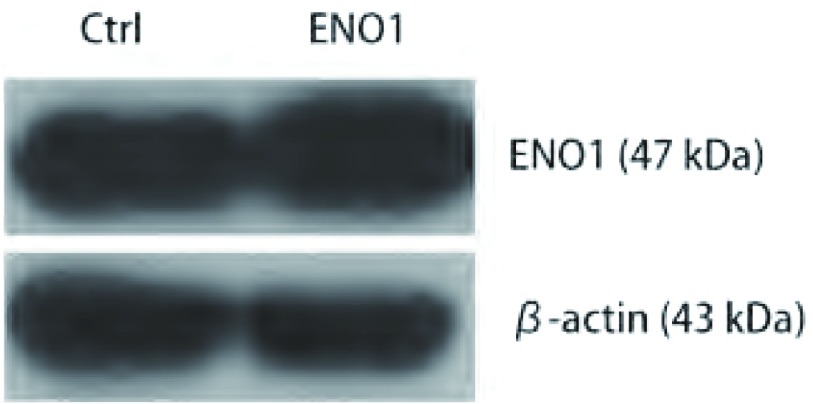
Western blot鉴定外源性*ENO1*基因在A549细胞中的过表达 The over-expression of exogenous ENO1 was examined by Western blot

### 2.2 过表达ENO1抑制细胞运动

划痕24 h、48 h和72 h后，A549-ENO1划痕仅愈合了19.6%、38.4%和63.4%，均低于对照组A549-Ctrl的34.5%、60.1%和85.9%（*P* < 0.05），表明ENO1过表达的A549细胞侧向运动能力明显下降（图 2A）。

**2:**
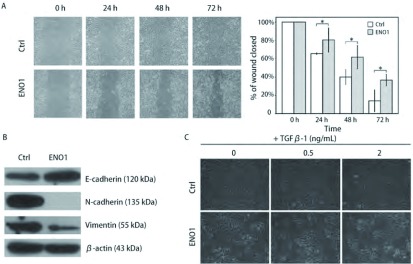
ENO1对A549细胞运动和EMT的抑制作用。A：划痕实验：在划痕后第24 h、48 h和72 h连续观察，过表达ENO1的A549细胞划痕恢复能力明显下降（^*^t检验，*P* < 0.05）。图表纵坐标表示划痕宽度相对0 h时划痕宽度比，横坐标表示时间；B：Western blot结果显示，相对于A549-Ctrl，A549-ENO1细胞中，E-cadherin表达明显增加，N-cadherin和Vimentin表达量明显降低；C：梯度TGF*β*-1诱导EMT实验：低剂量（0.5 ng/mL）处理A549-Ctrl细胞即可出现大量间质样细胞，而A549-ENO1细胞需要较高剂量（2 ng/mL）处理才能出现一定数量的间质样细胞。 ENO1 inhibits A549 cell mobility and EMT process. A: Wound-healing assay: a continuous observation showed that over-expression of ENO1 resulted in limitation of A549 mobility (^*^t-test, *P* < 0.05). In the chart, Y-axis represents the relative wound width and X-axis represents time period; B: Western blot assay: compared to A549-Ctrl, E-cadherin up-regulated in A549-ENO1, with down-regulation of N-cadherin and Vimentin; C: gradient dose of TGF*β*-1 inducing EMT: quantity of mesenchymal-like cells were obtained in A549-Ctrl population after low dose (0.5 ng/mL) of TGF*β*-1 inducing. Relative lower ratio of mesenchymal-like cells formed in A549-ENO1 population after high dose (2 ng/mL) of TGF*β*-1 treatment.

### 2.3 过表达ENO1抑制EMT过程

相比于对照组，A549-ENO1细胞中上皮样细胞标志物E-cadherin表达量明显上升，间质样细胞标志物N-cadherin和Vimentin的表达降低（图 2B）。TGFβ-1诱导EMT实验结果显示，较高浓度的TGFβ-1（2 ng/mL）处理24 h才能使A549-ENO1群体中出现较多的成纤维样细胞；对照组A549-Ctrl在低浓度TGFβ-1（0.5 ng/mL）处理24 h后即出现较多的成纤维样细胞（图 2C）。该结果说明ENO1过表达可以对TGFβ-1诱导A549细胞的EMT过程产生抑制效应。

### 2.4 ENO1抑制ERK磷酸化

相比于A549-Ctrl，A549-ENO1中磷酸化的ERK1/2水平明显下降（图 3A）。细胞血清饥饿4 h后，用终浓度为20 ng/mL的EGF同时处理1 h后发现，A549-ENO1细胞中磷酸化的ERK1/2明显低于对照组，而MEK1/2的磷酸化几乎不受影响（图 3B）。因此，我们推测ENO1可以通过抑制ERK1/2的磷酸化来抑制A549细胞的EMT。

**3:**
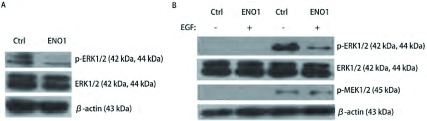
ENO1抑制A549细胞ERK1/2的磷酸化。A：同对照组相比A549-ENO1细胞中ERK1/2磷酸化水平明显受限；B：20 ng/mL的EGF处理细胞，A549-ENO1的ERK1/2磷酸化水平仍然明显低于对照组细胞，而MEK1/2磷酸化活化几乎不受影响。 ENO1 suppresses ERK1/2 phosphorylation in A549. Western blot result showed: A: Compared to A549-Ctrl group, ERK1/2 phosphorylation was inhibited by ENO1 over-expression; B: ERK1/2 phosphorylation level of A549-ENO1 was still lower than that of control group after EGF treatment, whereas MEK1/2 phosphorylation was almost not affected by ENO1 over-expression.

### 2.5 全长ENO1可以抑制EMT

野生型ENO1wt在第94和97位氨基酸残基对应的密码子是ATG，定点突变型ENO1m在对应位置为CTG（图 4A）。将野生型*ENO1*、突变型*ENO1*和空载质粒分别瞬时转染A549细胞48 h后，检测细胞中相关蛋白水平的变化，发现MBP-1表达没有明显的升高，并且突变型ENO1所产生的效应同野生型ENO1几乎完全相同（图 4B）。这表明全长ENO1蛋白可以阻碍EMT过程。

**4:**
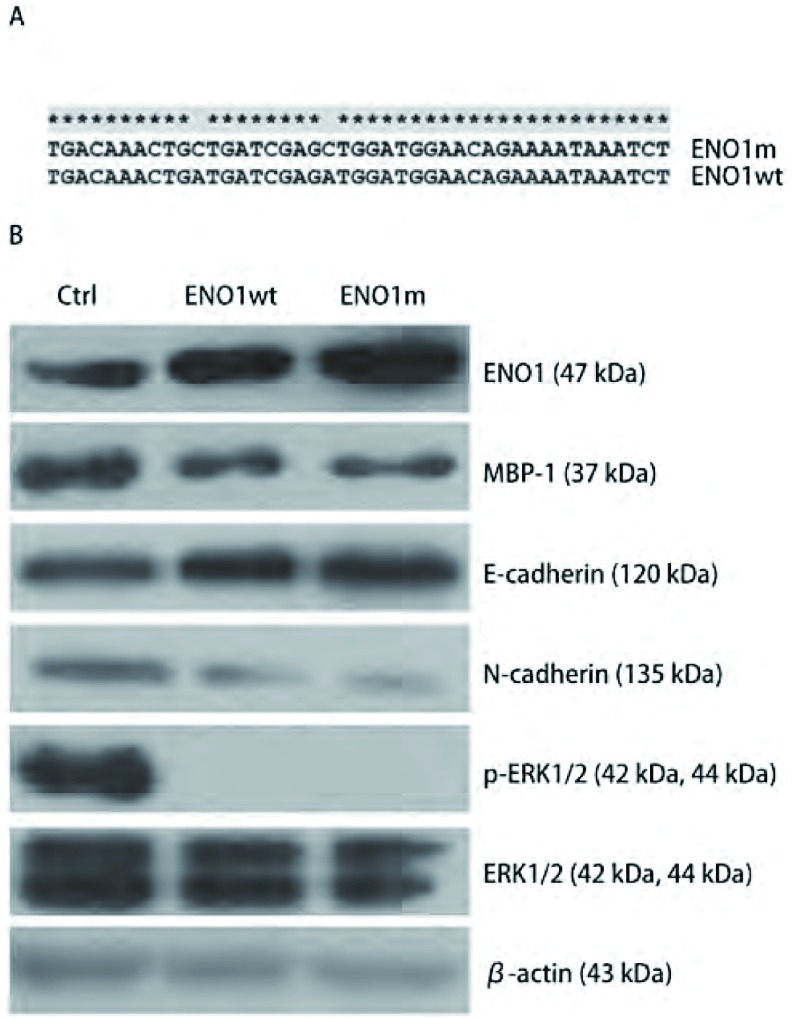
野生型和点突变ENO1具有相同作用。A：序列比对发现ENO1突变型存在两个ATG→CTG；B：Western blot检测显示出瞬时过表达野生型和突变型ENO1对E-cadherin、N-cadherin、p-ERK1/2变化影响是相似的。 Wild type and point mutant ENO1 have the same effect. A: Sequence comparison: two point mutation ATG->CTG existed in mutant ENO1; B: Western blot assay: highly similar effect on E-cadherin, N-cadherin and p-ERK1/2 appeared after transient overexpression of wild type and mutant ENO1 in A549.

## 3 讨论

本实验室利用新型蛋白质组研究体系建立了肺癌相关分泌/释放蛋白数据库，其中包含了ENO1在内的糖酵解途径关键酶^[21]^；并且检测到在NSCLC患者和正常对照组外周血中，ENO1蛋白水平具有明显差异，因此，我们推测ENO1可能同NSCLC的发展具有密切联系^[22]^。本实验则针对ENO1在NSCLC细胞的EMT过程中的生物学功能进行了初步研究。

本研究在A549细胞系内稳定过表达ENO1，发现EMT相关分子标志物的改变和抑制ERK1/2磷酸化的现象，并导致A549细胞的运动能力下降。对比图 2C中未添加TGFβ-1处理的细胞，我们发现过表达ENO1会引起A549细胞形态更趋近于上皮样细胞的变化，而对照组细胞更多地呈现出间质样细胞的梭形特征。根据这些现象，我们提出了ENO1通过抑制ERK1/2磷酸化来抑制EMT的假设。随后的TGFβ-1诱导EMT实验和EGF刺激ERK1/2活化的实验，均证实了这个假设。

在糖酵解过程中的很多酶都是多功能性的蛋白^[13]^。ENO1在不同的组织细胞中不同的生理和病理状态下，甚至是在不同的细胞亚定位均能够显示出不同的功能^[23]^。Hsu等^[20]^发现细胞核中的ENO1/MBP-1可以作为转录因子降低COX-2的转录水平，抑制胃癌细胞的EMT。对ENO1在NSCLC中抑制EMT的作用仍然没有解释清楚。

本研究结果显示全长ENO1蛋白本身具有抑制EMT的效应。为了排除MBP-1的干扰，我们构建了在ATG密码子处不产生选择性翻译MBP-1的突变型ENO1，并检测到过表达野生型ENO1和突变型ENO1在蛋白水平上对EMT相关分子标志物的改变作用几乎完全相同。而MBP-1对ERK信号通路的影响，则需要进一步的实验验证。

本研究不仅初步证实了ENO1的多功能性，为“糖酵解相关酶是多功能蛋白”的理论提供了新证据，而且也从影响细胞内信号传导的角度揭示ENO1抑制EMT的分子机理。经典的MAPK通路涉及到了Raf1→MEK1/2→ERK1/2的信号传递和放大过程^[24]^，磷酸化的ERK1/2激活转录因子c-Jun和c-Fos表达，进而促进*MMP-2*等基因表达，降解胞外基质，促进细胞运动^[25]^。当细胞接受MEK1/2特异性抑制剂U0126处理后，ERK1/2信号通路被阻断，弱化TGFβ诱导EMT的效果，表明ERK1/2通路的活化是细胞EMT过程的一个必要条件^[7]^。ERK1/2的第204/187位酪氨酸残基和MEK1/2第218/222位丝氨酸残基的磷酸化，分别指示了这两种激酶的活化状态，并且MEK1/2是目前已知唯一能够活化ERK1/2的激酶^[26, 27]^。基于这些条件，本实验分析了ENO1的过表达对ERK1/2信号通路的影响，进而发现了一个有趣的现象，即ENO1过表达引起活性状态的ERK1/2降低，而不影响MEK1/2活化。在另外一个研究^[18]^中，研究人员发现前列腺癌细胞中MBP-1可以通过同MEK5的直接相互作用抑制MEK5/BMK1的非经典MAPK信号通路。因此，我们推测在ERK1/2磷酸化/去磷酸化调节过程中，ENO1可能通过某种机制抑制了MEK1/2的催化活性，但不会影响MEK1/2的活化；也有可能是ENO1过表达，增强了PTPs和MKPs等磷酸酶活性，但这些推论需要更多的实验证据来支持。

## References

[1] Kochhar DM (1968). Studies of vitamin A-induced teratogenesis: effects on embryonic mesenchyme and epithelium, and on incorporation of H3-thymidine. Teratology.

[2] Acloque H, Adams MS, Fishwick K (2009). Epithelial-mesenchymal transitions: the importance of changing cell state in development and disease. J Clin Invest.

[3] Siegel R, Naishadham D, Jemal A (2013). Cancer statistics, 2013. CA Cancer J Clin.

[4] Denlinger CE, Ikonomidis JS, Reed CE (2010). Epithelial to mesenchymal transition: the doorway to metastasis in human lung cancers. J Thorac Cardiovasc Surg.

[5] Hugo H, Ackland ML, Blick T (2007). Epithelial--mesenchymal and mesenchymal--epithelial transitions in carcinoma progression. J Cell Physiol.

[6] Singh A, Settleman J (2010). EMT, cancer stem cells and drug resistance: an emerging axis of evil in the war on cancer. Oncogene.

[7] Xie L, Law BK, Chytil AM (2004). Activation of the Erk pathway is required for TGF-beta1-induced EMT *in vitro*. Neoplasia.

[8] Bhangu A, Wood G, Mirnezami A (2012). Epithelial mesenchymal transition in colorectal cancer: Seminal role in promoting disease progression and resistance to neoadjuvant therapy. Surg Oncol.

[9] Katoh M (2005). Epithelial-mesenchymal transition in gastric cancer (Review). Int J Oncol.

[10] Trimboli AJ, Fukino K, de Bruin A (2008). Direct evidence for epithelial-mesenchymal transitions in breast cancer. Cancer Res.

[11] Shih JY, Tsai MF, Chang TH (2005). Transcription repressor slug promotes carcinoma invasion and predicts outcome of patients with lung adenocarcinoma. Clin Cancer Res.

[12] Bayley JP, Devilee P (2012). The Warburg effect in 2012. Curr Opin Oncol.

[13] Kim JW, Dang CV (2005). Multifaceted roles of glycolytic enzymes. Trends Biochem Sci.

[14] Subramanian A, Miller DM (2000). Structural analysis of alpha-enolase. Mapping the functional domains involved in down-regulation of the *c-myc* protooncogene. J Biol Chem.

[15] Ray RB, Steele R, Seftor E (1995). Human breast carcinoma cells transfected with the gene encoding a c-myc promoter-binding protein (MBP-1) inhibits tumors in nude mice. Cancer Res.

[16] Chang YS, Wu W, Walsh G (2003). Enolase-alpha is frequently down-regulated in non-small cell lung cancer and predicts aggressive biological behavior. Clin Cancer Res.

[17] Takashima M, Kuramitsu Y, Yokoyama Y (2005). Overexpression of alpha enolase in hepatitis C virus-related hepatocellular carcinoma: association with tumor progression as determined by proteomic analysis. Proteomics.

[18] Ghosh AK, Steele R, Ray RB (2005). c-myc Promoter-binding protein 1 (MBP-1) regulates prostate cancer cell growth by inhibiting MAPK pathway. J Biol Chem.

[19] Ejeskar K, Krona C, Caren H (2005). Introduction of *in vitro* transcribed ENO1 mRNA into neuroblastoma cells induces cell death. BMC Cancer.

[20] Hsu KW, Hsieh RH, Wu CW (2009). MBP-1 suppresses growth and metastasis of gastric cancer cells through COX-2. Mol Biol Cell.

[21] Xiao T, Ying W, Li L (2005). An approach to studying lung cancer-related proteins in human blood. Mol Cell Proteomics.

[22] Zhang Y, Li M, Liu Y (2010). ENO1 protein levels in the tumor tissues and circulating plasma samples of non-small cell lung cancer patients. Zhongguo Fei Ai Za Zhi.

[23] Pancholi V (2001). Multifunctional alpha-enolase: its role in diseases. Cell Mol Life Sci.

[24] Sturgill TW (2008). MAP kinase: it's been longer than fifteen minutes. Biochem Biophys Res Commun.

[25] Chen PN, Hsieh YS, Chiou HL (2005). Silibinin inhibits cell invasion through inactivation of both PI3K-Akt and MAPK signaling pathways. Chem Biol Interact.

[26] Roskoski R Jr (2012). ERK1/2 MAP kinases: structure, function, and regulation. Pharmacol Res.

[27] Roskoski R Jr (2012). MEK1/2 dual-specificity protein kinases: structure and regulation. Biochem Biophys Res Commun.

